# Caffeine toxicity in zebrafish—Neurobehavioral changes, developmental defects, and oxidative stress: A review

**DOI:** 10.17305/bb.2025.13383

**Published:** 2025-12-22

**Authors:** Cătălina Ionescu, Petru-Fabian Lungu, Viorica Rarinca, Malina Visternicu, Alin Ciobica, Vasile Burlui, Cristina Albert, Mircea-Nicusor Nicoara, Gabriel-Ionut Plavan, Bogdan Novac, Bogdan Gurzu, Daniela Ivona Tomiţa

**Affiliations:** 1 Doctoral School of Biology, Faculty of Biology, “Alexandru Ioan Cuza” University of Iaşi, Iaşi, Romania; 2 “Ioan Haulica” Institute, Apollonia University, Iaşi, Romania; 3 Department of Biological and Morphofunctional Sciences, College of Medicine and Biological Science, Stefan cel Mare University of Suceava, Suceava, Romania; 4 Doctoral School of Geosciences, Faculty of Geography and Geology, “Alexandru Ioan Cuza” University of Iaşi, Iaşi, Romania; 5 Department of Biology, Faculty of Biology, Alexandru Ioan Cuza University of Iaşi, Iaşi, Romania; 6 “Olga Necrasov” Center, Department of Biomedical Research, Romanian Academy, Iaşi, Romania; 7 Academy of Romanian Scientists, Bucharest, Romania; 8 Preclinical Department, Apollonia University, Iaşi, Romania; 9 Grigore T. Popa University of Medicine and Pharmacy Iaşi, Iaşi, Romania

**Keywords:** Caffeine, zebrafish, neurobehavioral alterations, oxidative stress, developmental toxicity

## Abstract

Caffeine is one of the most widely consumed psychoactive stimulants, primarily functioning as a non-selective adenosine receptor antagonist. Its increasing detection in wastewater and surface waters reflects extensive anthropogenic use. This review synthesizes evidence from zebrafish (Danio rerio)—a genetically tractable vertebrate with rapidly developing external embryos—to assess the impact of caffeine exposure across environmentally relevant (ng–µg/L) and pharmacological/toxicological (mg/L and above) concentrations on early development and neurobehavior. Behavioral studies indicate dose- and stage-dependent alterations in locomotion, anxiety-like responses, memory performance, and sleep patterns, suggesting disruptions in neural circuitry and stress-axis regulation. Biochemical analyses frequently reveal oxidative imbalances characterized by increased reactive oxygen species and lipid peroxidation, alongside changes in antioxidant defenses (e.g., glutathione levels, superoxide dismutase [SOD] activity, and glutathione reductase activity). These findings support oxidative stress as a potential mechanistic hub, although a causal relationship has yet to be established. Embryonic exposure to caffeine is associated with developmental toxicity, including delayed hatching and concentration-dependent malformations such as edema, axial deformities, impaired angiogenesis, and neuromuscular defects at higher doses. However, cross-study comparisons are hindered by variations in units, exposure durations, and assay protocols. In summary, caffeine disrupts behavior, redox homeostasis, and developmental processes in zebrafish, highlighting the necessity for standardized methodologies to identify stage-specific vulnerabilities.

## Introduction

Caffeine is one of the most widely consumed psychoactive substances globally, naturally occurring in various foods and beverages. Following oral intake, caffeine is rapidly absorbed and distributed throughout the body, achieving peak plasma concentrations within 30–120 min [1]. The primary dietary sources include coffee (Robusta and Arabica), tea, chocolate, and soft drinks, with coffee being the predominant source for adults, while tea and sodas are more prevalent among adolescents [2]. In addition to its natural presence, caffeine can be synthetically produced, with no molecular differences from its natural form, and is commonly added to sodas and energy drinks. It is also incorporated into medications for headaches, colds, and allergies, utilized in cosmetic treatments, and recognized for its ergogenic effects in sports [3]. Caffeine is consumed not only for its stimulating effects but also for its pleasant taste, enhanced focus, and increased physical vitality [4].

As a staple in daily routines worldwide, caffeine has garnered increasing scientific interest as a bioactive molecule with both beneficial and adverse effects. Research indicates that caffeine influences multiple physiological systems, including the central nervous, urinary, digestive, and respiratory systems [3]. While these findings provide valuable insights into caffeine’s systemic activity in humans, they do not directly inform zebrafish-specific outcomes.

Some studies suggest that caffeine may alleviate anxiety and depression, while others demonstrate its potential to enhance learning and memory in tasks involving passive information presentation. Additionally, caffeine has been found to improve performance in tasks relying on working memory to some extent [5]. Furthermore, research indicates that caffeine significantly protects the brain against various forms of damage, such as neurotoxicity, seizures, and cognitive impairment, due to its rapid absorption in the gastrointestinal tract and distribution throughout the body, including the brain [6]. However, these findings serve solely as background information and are not considered evidence for zebrafish developmental outcomes.

The widespread use of caffeine has prompted scientific inquiry into its complex effects on various physiological systems, including the central nervous, cardiovascular, and digestive systems [7]. While moderate caffeine intake may enhance alertness, cognitive performance, and mood, excessive doses or chronic exposure have been linked to neurobehavioral disturbances, oxidative stress, and developmental toxicity [8–11].

Excessive caffeine consumption can lead to health issues such as sleep disturbances, anxiety, hypertension, and gastrointestinal discomfort, underscoring the importance of tailoring intake to individual tolerance [12]. Adverse effects of caffeine increase at high doses (9–13 mg/kg), although physical performance generally remains unaffected [13]. Intakes around 10–13 mg/kg have been associated with gastrointestinal disturbances, mental confusion, nervousness, difficulty concentrating, and sleep disruption in some individuals [14], while slightly lower doses (7–10 mg/kg) may cause chills, nausea, flushing, palpitations, headaches, and tremors [15]. Moderate doses (5–6 mg/kg) preserve ergogenic benefits while reducing, though not entirely eliminating, negative side effects and physiological responses. Caffeine doses of 200 mg or higher can lead to toxicosis, manifesting as restlessness, insomnia, muscle cramps, and periods of excessive alertness [16, 17].

In addition to its natural occurrence in coffee, tea, cacao, and other plants, caffeine is increasingly detected in the environment due to widespread human consumption in beverages, personal care products, and pharmaceuticals. It has been identified in treated wastewater (55–304 µg/L), groundwater (0.01–0.68 µg/L), drinking water (3.39 µg/L), rainwater (5.4 µg/L), rivers (0.01–49.6 µg/L), and lakes (0.02–174 µg/L), posing potential risks to aquatic ecosystems [18, 19]. Unlike humans, where caffeine exposure is primarily dietary, aquatic organisms encounter environmentally relevant concentrations through wastewater and surface waters. Consequently, zebrafish serve as a distinct model for ecotoxicological and developmental toxicity assessments. This raises concerns about caffeine’s effects on aquatic organisms, for which zebrafish provide a well-established and versatile model to explore both developmental toxicity and ecotoxicological outcomes. Due to their genetic similarity to humans, transparent embryos, and rapid development, zebrafish have become invaluable for investigating caffeine’s neurotoxic and teratogenic effects [20].

Zebrafish are small freshwater vertebrates native to the rivers of South Asia [21], typically measuring 3–4 cm in length and having a lifespan of about two years. Their externally developing, transparent embryos make them genetically accessible and highly versatile as a model organism. Over the past few decades, zebrafish have gained popularity in scientific research for studying a wide range of biological processes, particularly for investigating the neurotoxicity of drugs and environmental chemicals [22]. The advantages of using zebrafish as an *in vivo* model facilitate direct microscopic observation during early developmental stages. Additionally, their rapid growth and high fecundity enable high-throughput toxicity testing of multiple chemicals [23, 24].

Caffeine concentrations used in zebrafish studies vary widely, reflecting differences in experimental objectives. Lower concentrations, typically in the µg/L range, are considered environmentally relevant, corresponding to levels detected in surface waters and wastewater. Moderate concentrations, generally in the mg/L range, are often selected to simulate typical human consumption through beverages or foods. Higher concentrations, which may exceed 100 mg/L or 100 mg/kg in some studies, are primarily employed to investigate dose-dependent toxicity, anxiogenic effects, or developmental malformations.

Zebrafish studies have demonstrated that caffeine exposure can induce anxiety-like behaviors, disrupt sleep patterns, impair memory and neuromuscular development, and cause structural malformations in embryos, highlighting dose-dependent and stage-specific effects [25–28]. Caffeine exposure during early zebrafish development can result in various dose-dependent alterations, including cardiac and yolk sac edema, bent tails, spinal curvature, and impaired neuromuscular formation. These structural malformations are often accompanied by behavioral deficits such as reduced locomotor activity, disrupted anxiety-like responses, and impaired memory, emphasizing the sensitivity of developing embryos to oxidative stress and neurotransmitter imbalances induced by caffeine [29, 30].

**Figure 1.**
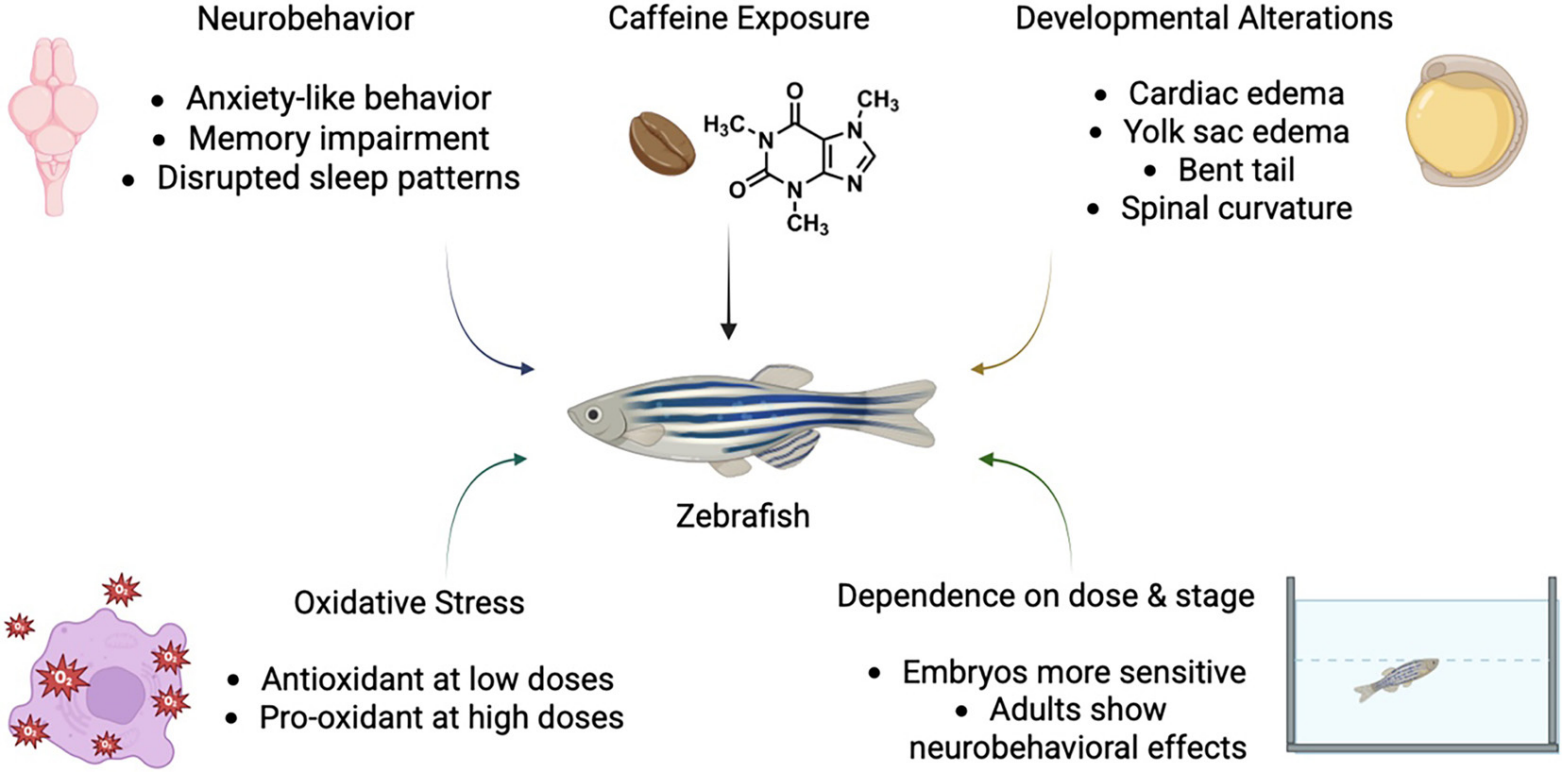
**Conceptual schematic summarizing reported associations between caffeine exposure and dose- and stage-dependent outcomes in zebrafish.** The diagram links caffeine exposure to neurobehavioral alterations and developmental alterations. Oxidative stress is highlighted as a contributing factor, depicted as antioxidant at low doses and pro-oxidant at high doses. The figure also emphasizes dependence on dose and developmental stage, indicating that embryos are more sensitive and that adults show neurobehavioral effects. *Created partially in BioRender.*

Mechanistically, caffeine exposure has been linked to oxidative stress, altered neurotransmitter signaling, and influences on developmental pathways, making zebrafish a versatile model for connecting molecular mechanisms to observable behavioral and morphological outcomes (Figure 1) [31, 32].

Although numerous studies report both beneficial and adverse effects of caffeine, the underlying mechanisms and dose-response relationships in zebrafish remain challenging to compare due to methodological variability across studies. This review posits that oxidative stress may represent a unifying mechanism linking caffeine’s diverse neurobehavioral and teratogenic effects; however, current research has yet to establish a clear causal relationship across different doses and developmental stages. Furthermore, inconsistencies in reported effects on anxiety and memory in zebrafish likely reflect variations in experimental conditions, doses, and developmental stages. This review aims to critically assess these variables, integrate findings, and propose a structured framework for interpreting caffeine’s dose-dependent neurobehavioral and developmental outcomes, providing guidance for future research directions.

### Caffeine: Pharmacology and mechanisms of action

Caffeine is the most widely consumed stimulant and psychoactive substance, naturally occurring in over 60 plant species, including coffee beans, cacao, and tea leaves. Known by various names such as guaranine, theine, or mateine, depending on its source, caffeine is chemically identified as C8H10N4O2, or 1,3,7-trimethylxanthine. This natural alkaloid belongs to the methylxanthine group and is often found alongside other bioactive compounds, such as polyphenols [6].

Structurally, caffeine is a heterocyclic organic compound with a purine base called xanthine, consisting of a pyrimidine ring linked to an imidazole ring. It is classified as a true alkaloid due to the presence of a heterocyclic nitrogen atom, although some researchers categorize it as a pseudo-alkaloid since its biosynthesis does not directly involve amino acids [33].

Caffeine has been demonstrated to enhance cognitive performance at low doses by blocking adenosine receptors, which leads to increased neuronal activity and the release of neurotransmitters such as dopamine and norepinephrine. This action results in improved reaction times and visual information processing [34]. Additionally, caffeine modulates neurotransmitter activity, particularly glutamate, which is essential for transmitting visual signals and further enhances reaction times [35].

Research indicates that cognitive benefits can be observed at doses as low as 0.18 mg/kg, with the dose–response relationship plateauing at higher intakes. Moreover, long-term caffeine consumption appears to induce tolerance, as habitual users often display diminished or absent cognitive effects [17].

Caffeine primarily functions as a non-selective antagonist of adenosine receptors, including subtypes A1, A2A, A2B, and A3. In the central nervous system, antagonism of A1 and A2A receptors reduces inhibitory signaling, which increases alertness, decreases fatigue, and enhances cognitive performance by facilitating neurotransmitter release, particularly dopamine and norepinephrine. These mechanisms also influence mood, attention, vigilance, and sleep regulation [36, 37]. Peripherally, caffeine impacts cardiovascular and renal functions: A1 receptor blockade in the heart increases heart rate, while antagonism of renal receptors enhances glomerular filtration and promotes diuresis. A2A receptor antagonism contributes to coronary vasodilation and can affect pain perception, which is relevant for migraine management [36]. Additionally, caffeine activates lipase to promote fat breakdown, modulates muscle contraction to enhance strength, and stimulates gastric acid secretion and gastrin release, thereby supporting digestive processes [38].

Epidemiological studies suggest that coffee intake is not associated with increased mortality; rather, modest inverse associations have been identified, correlating with reduced inflammation, improved endothelial function, and a lower risk of type 2 diabetes [38, 39]. Regular consumption appears to decrease susceptibility to low-density lipoprotein oxidation, protecting against atherosclerotic plaque formation, while phenolic compounds such as chlorogenic and ferulic acids provide significant antioxidant capacity. Protective effects have also been observed at the hepatic level, with reduced mortality in women suffering from liver disease or cirrhosis and a decreased risk of liver cancer. At the renal level, caffeine enhances natriuresis and diuresis through increased renal blood flow and reduced tubular sodium reabsorption, mechanisms comparable to thiazide diuretics [38, 40].

In addition to adenosine receptor antagonism, caffeine inhibits phosphodiesterase enzymes, leading to elevated intracellular levels of cyclic adenosine monophosphate (cAMP) and cyclic guanosine monophosphate. This elevation produces effects such as mild bronchodilation, lipolysis, and modulation of intracellular signaling pathways. Following absorption, caffeine is rapidly distributed throughout the body, primarily metabolized in the liver by cytochrome P450 enzymes, and excreted in the urine as various metabolites. Together, these central and peripheral actions underlie the cognitive-enhancing and physiological effects of caffeine in humans. Zebrafish, possessing conserved adenosine receptors and phosphodiesterase enzymes, serve as a versatile model to investigate how these mechanisms influence neurobehavioral function, oxidative stress, and development, bridging observations from human studies to experimental insights [2, 41].

## Zebrafish as a model organism in neurotoxicology: Assessing anxiety, memory and sleep alterations

The use of model organisms has been instrumental in advancing both biological and medical sciences. These species are extensively studied to elucidate specific biological processes, with the expectation that insights gained can be extrapolated to other organisms, including humans [42].

Caffeine exerts neuroactive effects primarily through the antagonism of adenosine receptors, which regulate neuronal excitability, sleep, and mood. In zebrafish, as in mammals, caffeine can induce anxiogenic behavior, disrupt sleep homeostasis, and alter circadian rhythms. These disruptions affect the hypothalamic-pituitary-interrenal (HPI) axis, leading to elevated cortisol levels that can have adaptive or maladaptive consequences based on their duration and intensity. Short-term caffeine exposure in zebrafish has been shown to increase anxiety, reduce exploratory behavior, elevate aggression, and impair HPI axis regulation. Prolonged exposure further impacts neurobehavioral functions, including swimming activity, social behavior, and memory, with high doses leading to cognitive deficits that may persist or emerge during withdrawal [8, 26, 43, 44].

### Anxiety

Anxiety is a neuropsychiatric disorder that significantly impacts quality of life and presents a persistent medical challenge. Research indicates that caffeine can induce anxiety-related behaviors, with high levels of caffeine exposure linked to increased anxiety levels in zebrafish [45].

Zebrafish exhibit pronounced anxiety-like behaviors in response to environmental stressors and display complex social interactions, making them an ideal model for studying neuropsychiatric disorders and pharmacological interventions [46]. They naturally demonstrate anxiety when introduced to novel environments. The Novel Tank Test (NTT) is commonly employed to assess this behavior by measuring the fish’s innate diving response. This test possesses good face and construct validity, as anxiolytic drugs reduce the diving response while anxiogenic compounds exacerbate it [47].

Studies in zebrafish have utilized a wide range of caffeine concentrations, categorized based on experimental intent:

**(1) Environmentally relevant exposures:** These concentrations range from ng/L to µg/L, consistent with levels detected in surface waters. Such levels (typically up to ∼10 µg/L) indicate trace contamination and are associated with subtle, chronic responses rather than overt toxicity [48, 49].

**(2) Pharmacological or toxicological paradigms:** These employ mg/L concentrations, often within the 100–1000 mg/L range, which are several orders of magnitude above environmental levels. Such exposures are used to investigate mechanistic responses, dose-dependent toxicity, or acute effects in zebrafish [50, 51].

Caffeine has been shown to induce anxiety-like behaviors in zebrafish across various developmental stages and experimental contexts. In larvae, exposure to laboratory doses of caffeine ranging from 100 to 300 mg/L altered locomotor activity in the Light-Dark Test (LDT), while exposure to 100–1000 mg/L provoked bradycardia and increased mortality [51]. Fontana and Parker (2022) validated the larval diving response (LDR) test in 7-day-old zebrafish larvae, demonstrating that increased time spent at the bottom of the tank under a 30-minute exposure paradigm reflects an anxiogenic response. Although this study used a 100 mg/L caffeine condition as part of the validation, it was not designed to investigate caffeine effects per se and serves solely to establish the behavioral assay [47]. Consistent with this paradigm, 7-day-old zebrafish larvae exposed to caffeine at concentrations up to 100 mg/L for 2 h in a thigmotaxis-based assay exhibited anxiety-like behavior, spending more time along the edges of the tank, supporting the interpretation that caffeine acts as an anxiogenic agent in larval models [52].

In adult zebrafish, short-term exposure to environmental concentrations of caffeine (0.5–300 µg/L) resulted in reduced exploratory behavior and heightened stress responses in the NTT. These findings suggest that even acute, low-level exposure can modulate anxiety-related behaviors, potentially through alterations in neurotransmitter signaling and stress-related pathways, highlighting possible ecological implications for aquatic populations [18]. Sex-specific differences have also been documented, with male and female zebrafish exhibiting distinct anxiety responses and physiological reactions to caffeine exposure, potentially due to variations in hormone levels, metabolism, and stress-axis regulation [19, 45]. High caffeine intake (100 mg/kg) has also been associated with oxidative stress-mediated anxiety, which can be mitigated by antioxidants such as alpha-tocopherol [53]. Environmental and social contexts further modulate these effects, with altered anxiety-like behaviors observed based on social stimuli. In a study involving adult zebrafish, exposure to caffeine at 25 mg/L (low dose) and 60 mg/L (moderate dose) for 10 min influenced anxiety-like behavior depending on social context. Anxiety-like effects were characterized by increased bottom-dwelling and freezing behavior, assessed using the NTT. Social behavior was evaluated separately using a social preference test (SPT), revealing reduced time spent near conspecifics at the higher dose. This study demonstrates that both anxiety and social responses to caffeine are influenced by dosage and social context [28]. In another study on adult zebrafish, caffeine exposure for 10 min induced anxiety-like behavior, with increased bottom-dwelling and freezing observed in the NTT. Social behavior was also impacted, as fish spent less time near conspecifics in the SPT, indicating decreased social interaction [46] (Table 1).

**Table 1:** Summary of caffeine-induced anxiety-like behaviors in zebrafish: Stages, doses, exposure durations, assessment methods, and references

**Zebrafish stage**	**Caffeine dose**	**Exposure route**	**Exposure period**	**Sample size (*n*)**	**Sex**	**Anxiety effects**	**Test(s) used**	**References**
Larvae (4 dpf)	100–300 mg/L (locomotor effects), 100–1000 mg/L (bradycardia and mortality) (≈515–5150 µM)	Immersion	4 h	Not reported	Not reported	- Altered locomotor activity in LD cycles - Bradycardia - Higher mortality	LDT	[51]
Larvae (7 dpf)	10–100 mg/L (≈52–515 µM)	Immersion	2 h	300 (80 per group)	Not reported	- Increased edge-preference (thigmotaxis) - Reduced bottom-dwelling avoidance - Altered locomotor activity	Thigmotaxis-based “edge preference” assay	[47]
Adults	0.5, 1.5 and 300 µg/L (≈0.0026, 0.0077, and 1.54 µM)	Immersion	7 days	12 per group	Mixed (males and females)	- Reduced exploratory behavior - Enhanced stress responses	NTT	[18]
	100 mg/L (≈515 µM)	Immersion	6 min	Not reported	Mixed (males + females)	- Males showed heightened anxiety-like behaviors in response to caffeine - Females exhibited stronger alarm responses to conspecific alarm substance and aversion to predator sight	NTT	[19]
	0.3 mg/L to 600 mg/L (≈1.54–3089 µM)	Immersion	Acute	Not reported	Mixed (males + females)	- Males exhibited more erratic and chaotic swimming patterns, reflecting stress-induced anxiety behaviors - Females showed a longer latency to explore the upper zone of the tank, and freezing behavior, indicating heightened anxiety-like responses	NTT	[45]
	100 mg/kg (systemic dose)	Dietary	30 min	166 (10–12 per group)	Not reported	- Anxiety-like behaviors such as increased thigmotaxis (preference for the periphery of the tank) - Freezing behavior - Erratic swimming	NTT	[53]
	25 mg/L and 60 mg/L (≈129–309 µM)	Immersion	10 min	Not reported	Mixed (males + females)	- Increased bottom-dwelling - Increased freezing behavior	NTT; SPT	[28]
	20 and 100 mg/L (≈103–515 µM)	Immersion	10 min	Not reported	Not reported	- Mild to pronounced increase in bottom-dwelling and freezing behavior, indicating heightened anxiety - Reduced time spent near conspecifics, indicating decreased social interaction	NTT; SPT	[46]

Abbreviations: dpf: Days post-fertilization; LD: Light–dark (cycles); LDT: Light–dark test; NTT: Novel tank test; SPT: Social preference test; LDR: Larval diving response.

Collectively, these findings suggest that caffeine acts as an anxiogenic agent in zebrafish, likely by modulating neurotransmitter systems such as dopamine, norepinephrine, and glutamate, as well as altering stress-axis activity. The effects are dose-dependent and vary with developmental stage, as evidenced by increased bottom-dwelling and freezing behavior in the NTT and reduced social interaction in the SPT.

### Memory

Zebrafish exhibit strong neuroanatomical and neurotransmitter similarities to humans, including homologues of the hippocampus, amygdala, and isocortex, as well as conserved glutamatergic, GABAergic, and cholinergic signaling. These features support complex neurobehaviors, including learning, memory retention, spatial and object recognition, and fear responses. Consequently, zebrafish serve as a valuable vertebrate model for studying memory and cognitive function, including research on cognitive decline and drug discovery [54].

The impact of caffeine on memory in zebrafish is not uniform, varying according to dose, developmental stage, and experimental conditions. For instance, juvenile zebrafish exposed to relatively high caffeine concentrations (20 and 50 mg/dL, equivalent to 200 and 500 mg/L) under unpredictable chronic stress for three days exhibited impairments in working memory when tested in the T-maze, indicating that caffeine may exacerbate stress-induced cognitive deficits [55]. In contrast, adult zebrafish exposed for 14 days to lower caffeine concentrations (10 and 50 mg/L) demonstrated improved task performance, with the 10 mg/L group reaching the reward target faster and spending more time near it. However, these effects were associated with increased locomotor activity, suggesting that enhanced performance may reflect heightened arousal and attention rather than pure improvements in memory [56].

In summary, these findings reveal that caffeine can exert both detrimental and beneficial effects on zebrafish cognition. Negative outcomes are more likely at higher doses, during developmental stages, under stress conditions, and with short exposure durations, while beneficial outcomes appear at lower doses in adults during longer exposure periods [18]. Methodological differences, such as dose units, stress paradigms, and behavioral tasks, likely contribute to the apparent discrepancies [57]. Mechanistically, the biphasic effects of caffeine may reflect adenosine receptor antagonism, where low doses enhance attention and vigilance, whereas higher doses disrupt glutamatergic and GABAergic balance, stress hormone regulation, and sleep-related processes critical for memory consolidation [58].

### Sleep

Sleep is a fundamental and conserved characteristic of animal life, primarily facilitating brain functions such as energy replenishment and memory consolidation. Its timing and intensity are regulated by circadian rhythms and homeostatic sleep pressure, which reflect prior neuronal activity [59].

Research in zebrafish has demonstrated that increased neuronal activity, induced by stimulants like caffeine, leads to rebound sleep, independent of prior wake duration or physical activity. This suggests a close relationship between sleep need and overall brain activity [60]. The noradrenergic system, particularly the locus coeruleus, plays a critical role in maintaining wakefulness and modulating sleep pressure, with fluctuations in its activity affecting both arousal and subsequent sleep [61]. Animal models, including zebrafish, have significantly advanced our understanding of sleep mechanisms. Unlike nocturnal rodents, zebrafish exhibit diurnal sleep patterns akin to those of humans [62]. Their pineal gland becomes fully developed by 19–20 h post-fertilization (hpf) and produces melatonin at night under circadian regulation [63]. Zebrafish larvae display complex sleep behaviors as early as 4 days post-fertilization, and their small size facilitates detailed monitoring using videography in a 96-well plate format. Given caffeine’s established effects on sleep in humans, one study explored its impact on sleep and behavior in zebrafish larvae. Exposure to caffeine at concentrations of 31.25–120 µM for 48 h disrupted normal sleep patterns, significantly reducing total sleep time and sleep efficiency. These findings align with observations in adult zebrafish and other model systems, emphasizing the potential relevance of caffeine-induced sleep disturbances for understanding human sleep regulation [27].

Caffeine has also been shown to counteract cognitive deficits associated with sleep deprivation in adult zebrafish. This effect is mediated through the activation of protein kinase A (PKA), which regulates O-linked β-N-acetylglucosamine cycling, highlighting a molecular pathway by which caffeine may alleviate sleep-related memory impairments [64].

## Developmental alterations induced by caffeine in zebrafish

The neurobehavioral deficits observed in adult zebrafish may stem from subtle developmental alterations induced by caffeine exposure during embryogenesis, where it disrupts key morphological processes [65]. Investigating these developmental changes in zebrafish is essential due to their rapid embryonic development, genetic resemblance to humans, and transparent embryos that allow for precise observation of morphological, cardiovascular, and neurobehavioral effects, thereby making them an ideal model for assessing the potential toxicity and teratogenicity of various compounds [24, 66].

Previous studies have indicated that caffeine can induce teratogenic and long-term neurodevelopmental effects in zebrafish embryos through oxidative stress-mediated apoptosis. In a study by Felix et al., embryos (∼2 hpf) were exposed to 0.5 mM caffeine, either alone or in conjunction with 24-epibrassinolide (24-EPI) at concentrations of 0.01, 0.1, and 1 µM, for 96 h. Caffeine exposure alone significantly increased developmental malformations, including edema and tail curvature, as well as locomotor deficits characterized by decreased speed and distance traveled, alongside disrupted anxiety-like and avoidance behaviors [25]. High doses of caffeine have been shown to induce significant developmental toxicity in zebrafish embryos and larvae. Exposure during the 2–24 hpf window to concentrations ranging from 10 µM to 500 µM resulted in dose-dependent developmental defects, including low morphological scores affecting the notochord and heart, general malformations, diminished normal phenotypes, disrupted sleep rhythms, and altered locomotor activity. Wei et al. found that moderate concentrations (31.25–125 µM) resulted in minimal developmental defects, high survival rates, normal angiogenesis, and no inflammatory response, indicating a relatively safe range for behavioral assays. In contrast, higher concentrations (250–500 µM) caused pronounced developmental defects and low morphological scores (notochord, heart), while very high concentrations (1000–2000 µM) induced severe developmental defects with high mortality. These findings suggest that caffeine exposure during early life stages can severely impact structural development in zebrafish, while behavioral effects, such as disrupted sleep rhythms and altered locomotor activity, can be assessed safely within the 31.25–125 µM range, underscoring the importance of carefully considering dose and exposure duration in neurodevelopmental and behavioral studies [27].

Furthermore, exposure of zebrafish embryos to caffeine at concentrations of 250–350 ppm resulted in significant developmental alterations, particularly affecting vascular formation, as noted by Yeh et al. in their investigation. The embryos exhibited abnormal development of intersegmental vessels, dorsal longitudinal anastomotic vessels, and subintestinal vein sprouting, indicating impaired angiogenesis [29]. Additional findings suggested that exposure to caffeine at concentrations ranging from 17.5 to 150 mg/L led to significant neuromuscular and developmental alterations. At the highest concentration (150 mg/L), embryos demonstrated reduced body length (2.67 ± 0.03 mm compared to 3.26 ± 0.01 mm in controls) and a marked decrease in touch-induced movement, dropping from 9.93 ± 0.77 in controls to 0.10 ± 0.06. Immunostaining revealed misalignment of muscle fibers and defects in primary and secondary motor axon projections, indicating impaired neuromuscular development, as reported in the study by Chen et al. These results highlight that caffeine at moderate to high doses disrupts normal motor function and neuromuscular formation in zebrafish embryos, emphasizing its potential impact on early motor behavior and structural development [67]. Moreover, caffeine exposure has been shown to affect early developmental processes in zebrafish larvae. In the 2011 study by Chakraborty et al., caffeine treatment significantly increased heart rate, reaching 125–140 beats per minute, while simultaneously reducing the expression of vascular endothelial growth factor, a critical regulator of angiogenesis. These findings suggest that high doses of caffeine can disrupt normal vascular development and potentially lead to developmental defects in zebrafish embryos, highlighting the sensitivity of early developmental stages to chemical exposure [68] (Table 2).

**Table 2:** Summary of caffeine-induced developmental effects in zebrafish embryos and larvae: Stages, doses, exposure durations, developmental defects, and references

**Zebrafish stage**	**Caffeine dose**	**Exposure route**	**Exposure period**	**Sample size (*n*)**	**Sex**	**Developmental alterations**	**References**
Embryos (2 hpf)	0.5 mM (≈500 µM)	Immersion	96 h	Not reported	Not reported	- Increased malformations including edema, tail curvature - Locomotor deficits	[25]
Embryos (2 hpf)	31.25–2000 µM	Immersion	24–72 hpf	30 per concentration (developmental assays); 24 per group (behavioral assays)	Not reported	- Minimal developmental defects, high survival rates, normal angiogenesis and no inflammatory response (31.25–125 µM) - Pronounced developmental defects, low morphological scores (notochord, heart) (250–500 µM) - Severe developmental defects, high mortality (1000–2000 µM)	[27]
Embryos	250–350 ppm (≈1288–1802 µM)	Immersion	Not reported	Not reported	Not reported	- Abnormal intersegmental vessels - Dorsal longitudinal anastomotic vessels - Subintestinal vein sprouting	[29]
Embryos	17.5, 35, 50, 100, and 150 mg/L (≈90–772 µM)	Immersion	Not reported	Not reported	Not reported	- Reduced body length - Decreased touch-induced movement - Misaligned muscle fibers - Defective motor axon projections	[67]
Larvae	10, 20, 50, and 100 µg/mL (≈52–515 µM)	Immersion	Not reported	Not reported	Not reported	- Increased heart rate (125–140 bpm) - Potential vascular developmental defects	[68]

Abbreviations: hpf: Hours post-fertilization; bpm: Beats per minute.

## Oxidative stress and antioxidant responses

Oxidative stress occurs when the production of reactive species surpasses the capacity of cellular defenses to neutralize them. Reactive oxygen species (ROS), such as superoxide anions and hydrogen peroxide, can generate highly reactive hydroxyl radicals in the presence of transition metals. Additionally, interactions between superoxide and nitric oxide produce reactive nitrogen species, including peroxynitrite, which can also form hydroxyl radicals [36].

Caffeine influences oxidative stress in the brain by modulating ROS and neurotransmitter systems, particularly glutamate. While oxidative stress is known to contribute to neurobehavioral alterations, its role as a mechanism underlying caffeine-induced behavioral changes remains to be fully elucidated [53].

Coffee is a primary dietary source of antioxidant compounds that neutralize ROS, the main contributors to oxidative stress [69]. Although caffeine is often acknowledged for its antioxidant properties, some studies indicate it can act as a prooxidant under certain conditions. For instance, Gülçin (2008) demonstrated that caffeine promoted linoleic acid peroxidation in emulsions at concentrations of 15, 30, and 45 µg/mL, resulting in oxidation levels of 32.5%, 48.9%, and 54.3%, respectively. These findings support the notion that caffeine may contribute to oxidative processes rather than solely neutralizing free radicals, depending on the environment and concentration. This dual behavior underscores the complexity of caffeine’s biological effects and suggests that its role in oxidative stress may be context-dependent [70].

Research on coffee consumption and its antioxidant effects has yielded mixed results. The studies mentioned previously were conducted in humans, examining the impact of coffee consumption on plasma antioxidant capacity and endogenous antioxidant enzymes. Given the limited research on these effects in zebrafish, investigating caffeine’s influence on antioxidant defenses in this model is crucial for understanding underlying molecular mechanisms and developmental implications. Some studies have shown that coffee intake can significantly increase plasma antioxidant capacity [71–74]. For example, a single serving of 200–400 mL of coffee raised plasma antioxidant markers by 2%–7% [72, 73], although long-term interventions often displayed inconsistent effects [71, 74]. Chronic trials have also examined endogenous antioxidant enzymes such as SOD, catalase (CAT), glutathione peroxidase (GPx), glutathione reductase (GSR), and glutathione S-transferases (GSTs), with some studies documenting increases of up to 75% for SOD and around 60% for GPx [74]. In contrast, other studies found reduced enzyme activity [75], and results regarding glutathione (GSH) were variable, with some studies reporting increases [76, 77], while others found no effect [78, 79]. Overall, coffee can enhance antioxidant defenses, but the extent of the effect depends on coffee type, dosage, and study design.

**Table 3:** Summary of research examining the impact of caffeine on oxidative stress and antioxidant responses in zebrafish

**Zebrafish stage**	**Caffeine dose**	**Exposure route**	**Exposure time**	**Sample size (*n*)**	**Sex**	**Oxidative stress marker**	**Oxidative/Antioxidant effects**	**References**
Embryos	100 µM	Immersion	24-96 hpf	Not reported	Not reported	HSP70 ↑ Cyclin G1 ↑ Bax/Bcl-2 ratio ↑	↑ Gene expression related to cell damage and apoptosis; mitochondrial dysfunction - Oxidative stress during early development	[80]
Adult	0.16, 0.42, 1.09, 2.84, 7.40, 19.23, and 50 µg/L (≈0.0008–0.26 µM)	Immersion	28 days	15 per group	Not reported	SOD ↑ GRed ↑ GSH ↓ AChE ↓	↑ Antioxidant enzymes (SOD, GRed), ↓ glutathione, lipid peroxidation unchanged metabolic alterations ( ↑ lipid content); High doses (19.23 and 50 µg/L) ↓ acetylcholinesterase, suggesting neurotoxicity	[81]
Adult	100 mg/kg (systemic dose)	Dietary	Not reported	166 (10-12 per group)	Not reported	MDA ↑	↑ Lipid peroxidation in brain - Antioxidant alpha-tocopherol prevented oxidative stress	[53]

↑ up-regulation/increase; ↓ down-regulation/decrease. Abbreviations: hpf: Hours post-fertilization; HSP70: Heat shock protein 70; SOD: Superoxide dismutase; GRed: Glutathione reductase; GSH: Reduced glutathione; AChE: Acetylcholinesterase; MDA: Malondialdehyde; LDH: Lactate dehydrogenase.

Building on these human studies, several investigations have examined the effects of caffeine on oxidative stress and antioxidant defenses in zebrafish, providing experimental insights into molecular impacts. In a study by Abdelkader et al. (2013), prolonged caffeine exposure induced oxidative stress in zebrafish embryos, evidenced by increased gene expression related to cell damage and apoptosis, as well as mitochondrial dysfunction. These findings suggest that caffeine exposure during early developmental stages can compromise cellular integrity and function in zebrafish embryos [80]. Additionally, another study exposed zebrafish to caffeine concentrations ranging from 0.16 to 50 µg/L for 28 days, revealing increased activity of antioxidant enzymes such as SOD and GSR, alongside a reduction in glutathione levels. Lipid peroxidation remained unchanged, while metabolic alterations included decreased LDH activity and higher lipid content. At the highest doses (19.23 and 50 µg/L), a reduction in acetylcholinesterase activity suggested possible neurotoxic effects. These results indicate that caffeine exposure can disrupt oxidative balance and metabolic function in zebrafish, even at low, environmentally relevant concentrations, highlighting the importance of dosage and developmental stage in determining its toxicological impact [81]. Another study demonstrated that high doses of caffeine induce oxidative stress in the brain, evidenced by a significant increase in lipid peroxidation, as measured by elevated malondialdehyde (MDA) levels. Interestingly, treatment with the antioxidant alpha-tocopherol prevented this biochemical alteration and reduced anxiety-like behaviors induced by caffeine, suggesting that oxidative stress plays a vital role in mediating the neurobehavioral effects of caffeine in zebrafish [53] (Table 3).

While our findings indicate that caffeine exposure in zebrafish induces a range of developmental abnormalities and oxidative stress responses, the molecular mechanisms underlying these effects remain only partially understood. To better contextualize the malformations and redox imbalances observed in our model, further research is needed in mechanistic domains such as oxidative stress pathways, mitochondrial dysfunction, intrinsic and extrinsic apoptotic signaling, and the cAMP–PKA–cAMP response element-binding protein (CREB) axis. These molecular processes may elucidate how caffeine disrupts early development and cellular homeostasis in zebrafish and potentially in other vertebrate systems.

Some cellular experiments suggest that caffeine promotes apoptosis through processes linked to oxidative imbalance. For instance, caffeine exposure has been reported to disrupt key survival pathways such as Ras, Akt, and extracellular signal-regulated kinase (ERK), with these alterations being mitigated by antioxidant treatment or by inhibiting proteasomal activity. Such findings indicate a contribution of oxidative stress and protein degradation systems to caffeine-induced apoptotic responses [82].

Conversely, other evidence suggests that caffeine may enhance mitochondrial performance in contexts of mitochondrial deficiency. Certain models demonstrate that caffeine administration can improve respiratory capacity, ATP synthesis, and the respiratory control ratio (RCR) [83]. Similarly, renal cell studies have documented increases in mitochondrial membrane potential and ATP levels following caffeine exposure, implying that mitochondrial energetics may be modulated in a cell-specific manner [84].

Caffeine’s influence on second-messenger pathways, particularly those governed by cAMP–PKA, also warrants further exploration. In endothelial cells, caffeine has been shown to elevate cAMP, activate PKA, and trigger AMPK-dependent mitochondrial fragmentation, a process associated with altered cellular dynamics and mitochondrial function [85].

These observations suggest that cAMP–PKA–CREB signaling serves as a pivotal mediator linking caffeine exposure to subsequent metabolic or apoptotic changes.

## Conclusion

Evidence from zebrafish studies demonstrates that caffeine produces complex, dose-dependent neurobehavioral effects, particularly regarding anxiety, memory, and sleep. At low doses, caffeine may enhance attention and memory performance; however, higher concentrations consistently elicit anxiety-like behaviors, reduce exploratory activity, and disrupt normal social interactions. Sleep regulation is significantly impacted, with larval zebrafish exposed to caffeine exhibiting reduced total sleep time and efficiency. In adult zebrafish, caffeine can temporarily alleviate memory impairments induced by sleep deprivation through molecular pathways such as PKA activation. Collectively, these findings reinforce the central thesis of this review: caffeine’s effects are significantly influenced by dose and developmental stage, with oxidative stress frequently implicated as a potential unifying mechanism, underscoring both its potential benefits and toxic risks throughout development.

In addition to behavioral outcomes, caffeine exposure during early zebrafish development leads to distinct morphological and structural alterations. Embryos and larvae exposed to moderate and high doses exhibit dose-dependent malformations, including cardiac and yolk sac edema, spinal curvature, impaired angiogenesis, and neuromuscular defects. These alterations compromise survival and result in long-term functional deficits, such as reduced locomotor activity and impaired anxiety or avoidance behaviors. Although several studies suggest that these teratogenic outcomes may involve caffeine-induced mitochondrial dysfunction and increased ROS production, oxidative stress should be regarded as a plausible but not yet definitively proven unifying mechanism. These findings highlight the heightened vulnerability of embryonic and larval stages to caffeine and position zebrafish as an effective model for developmental neurotoxicology.

Mechanistically, many of these effects are mediated through oxidative stress pathways. Caffeine functions as both an antioxidant and a prooxidant, with zebrafish studies indicating upregulation of oxidative stress-related genes, mitochondrial dysfunction, lipid peroxidation, and reductions in glutathione levels. Concurrently, the compensatory activation of antioxidant enzymes such as SOD and GRed suggests an adaptive response to redox imbalance. Notably, antioxidant supplementation, such as alpha-tocopherol, has been shown to alleviate oxidative stress and caffeine-induced anxiety behaviors, supporting—but not conclusively establishing—a mechanistic link between redox regulation and neurobehavioral outcomes. Overall, zebrafish studies indicate that caffeine’s influence on anxiety, memory, sleep, and development is intricately connected to its oxidative stress-modulating properties, emphasizing the importance of dose, exposure duration, and developmental stage in determining its beneficial vs detrimental effects.

Despite significant advancements, several critical questions remain unresolved. While associations between oxidative stress and anxiety, memory, sleep, and teratogenic outcomes are increasingly documented, the specific neural circuits, cell populations, and molecular targets most vulnerable to caffeine in zebrafish remain largely unexplored. The long-term consequences of early-life caffeine exposure, including potential persistent behavioral and physiological deficits, are poorly understood. Additionally, interactions with antioxidant interventions such as N-acetylcysteine (NAC), sex-specific responses, and the distinction between environmentally relevant vs pharmacological dosing require further clarification.

Despite the increasing number of studies investigating caffeine’s effects in zebrafish, several limitations hinder the generalizability and comparability of existing findings.

First, assay heterogeneity poses a significant challenge: behavioral, biochemical, and developmental assays vary widely in sensitivity, endpoints, and methodological execution, complicating direct comparisons.

Second, dose selection and exposure paradigms differ greatly across studies, encompassing environmentally relevant concentrations to pharmacological or supra-environmental doses, and often vary in exposure duration, renewal frequency, and developmental timing.

Third, sex is seldom reported or controlled for, particularly in behavioral assays, despite well-documented sex-specific differences in stress- and anxiety-related responses.

Fourth, social and environmental contexts, including housing density, group vs individual testing, and tank conditions, can significantly influence zebrafish behavior; however, this is inconsistently documented.

Finally, the field may be affected by reporting and publication biases, wherein positive or dose-dependent effects are more likely to be published compared to null or contradictory results. Collectively, these factors underscore the necessity for more standardized experimental designs and transparent reporting practices to enhance reproducibility and clarify caffeine’s developmental and neurobehavioral effects across studies.

Future research should employ mechanistic and integrative approaches to elucidate caffeine’s effects. For example, CRISPR/Cas9 and other genetic tools could target key antioxidant-related genes (e.g., Nrf2) to directly assess whether oxidative stress plays a causal role in caffeine-induced teratogenesis. Longitudinal studies tracking zebrafish from embryogenesis to adulthood are essential to determine the persistence and reversibility of neurobehavioral and developmental deficits. Standardized, dose-dependent experimental designs integrating behavioral, morphological, and molecular endpoints will help differentiate subtle environmentally relevant effects from overt pharmacological or toxic outcomes.
